# Supplementary material for: Tick cells as a tractable arthropod model for studying Orientia tsutsugamushi
Source: Appl Environ Microbiol. 2026 Jun 12;92(7):e02540-25. doi: 10.1128/aem.02540-25 (PMC13390357; doi:10.1128/aem.02540-25)
Supplement: Supplemental material — Figures S1 to S3. [file aem.02540-25-s0001.pdf]

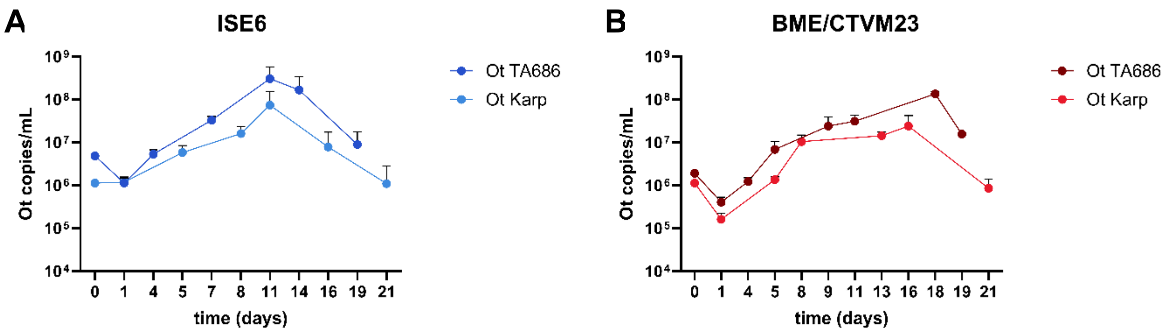

Supplementary Figure 1. *Orientia tsutsugamushi* strains TA686 and Karp grow in tick cell lines. A) Graphs showing bacterial copy number per mL of the *Ot* strains TA686 and Karp in ISE6 at 32 °C. B) Bacterial copy number of the *Ot* strains TA686 and Karp in BME/CTVM23. Bacterial load was measured by qPCR using primers against the conserved single-copy bacterial gene *tsa47*. Graph shows mean and standard deviation taken from three independent biological replicates. Statistical analysis was carried out using two-way ANOVA and showed no significant difference in growth between *Ot* TA686 and Karp ($p>0.05$; ns).

5 µm.

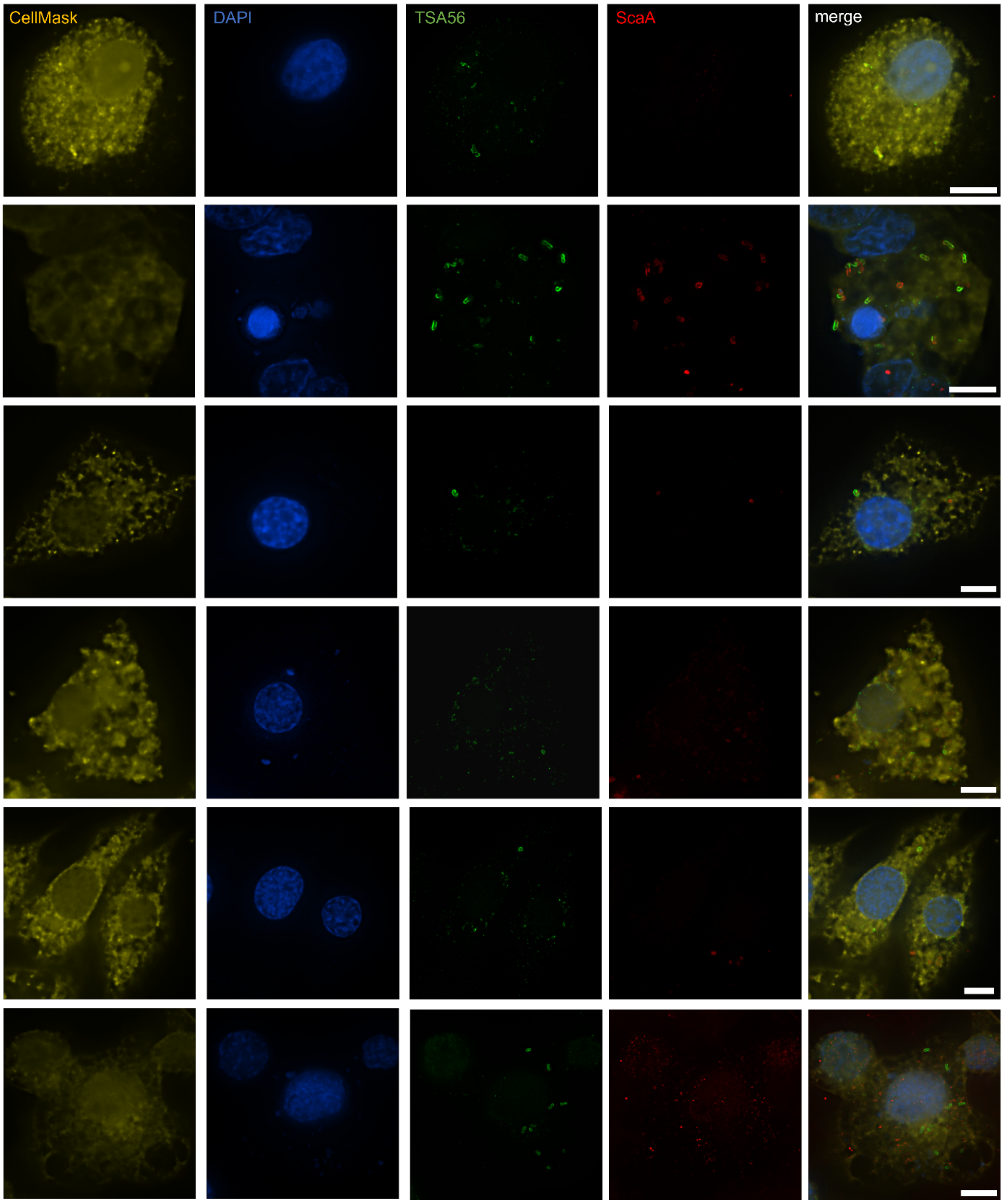

Supplementary Figure 2. *Orientia tsutsugamushi* strain Karp at day one post infection in ISE6 cells showing that bacteria were dispersed throughout the cytoplasm in the host cell. The bacteria expressed the outer membrane proteins TSA56, ScaA. Cytosol and nuclei were stained with CellMask and DAPI, respectively. Scale bars represent 5 µm.

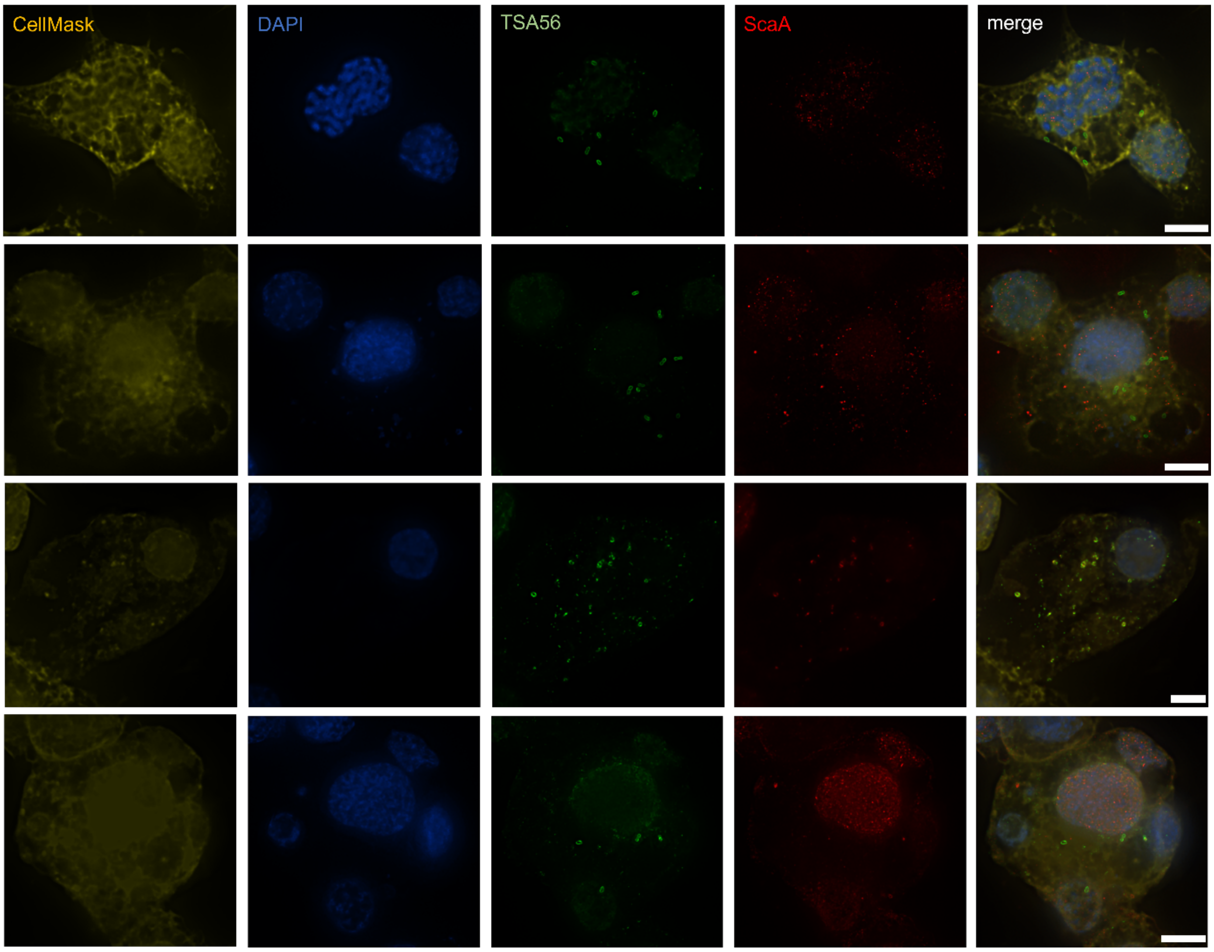

Supplementary Figure 3. *Orientia tsutsugamushi* strain TA686 at day one post infection in ISE6 cells showing that bacteria were dispersed throughout the cytoplasm in the host cell. The bacteria expressed the outer membrane proteins TSA56, ScaA. Cytosol and nuclei were stained with CellMask and DAPI, respectively. Scale bars represent 5 µm.
